# The impact of diets containing *Hermetia illucens* meal on the growth, intestinal health, and microbiota of gilthead seabream (*Sparus aurata*)

**DOI:** 10.1007/s10695-024-01314-9

**Published:** 2024-02-22

**Authors:** Simona Rimoldi, Ambra Rita Di Rosa, Marianna Oteri, Biagina Chiofalo, Imam Hasan, Marco Saroglia, Genciana Terova

**Affiliations:** 1 Department of Biotechnology and Life Sciences, University of Insubria, Via J.H. Dunant, 3, 21100 Varese, Italy; 2 Department of Veterinary Sciences, University of Messina, Messina, Italy

**Keywords:** Aquaculture; Gilthead seabream, Gut microbiota, Intestinal health; *Hermetia illucens*

## Abstract

The present study investigated the effect of replacing fishmeal (FM) with insect meal of *Hermetia illucens* (HI) in the diet of *Sparus aurata* farmed inshore on growth, gut health, and microbiota composition. Two isolipidic (18% as fed) and isoproteic (42% as fed) diets were tested at the farm scale: a control diet without HI meal and an experimental diet with 11% HI meal replacing FM. At the end of the 25-week feeding trial, final body weight, specific growth rate, feed conversion rate, and hepatosomatic index were not affected by the diet. Gross morphology of the gastrointestinal tract and the liver was unchanged and showed no obvious signs of inflammation. High-throughput sequencing of 16S rRNA gene amplicons (MiSeq platform, Illumina) used to characterize the gut microbial community profile showed that Proteobacteria, Fusobacteria, and Firmicutes were the dominant phyla of the gut microbiota of gilthead seabream, regardless of diet. Dietary inclusion of HI meal altered the gut microbiota by significantly decreasing the abundance of *Cetobacterium* and increasing the relative abundance of the *Oceanobacillus* and *Paenibacillus* genera. Our results clearly indicate that the inclusion of HI meal as an alternative animal protein source positively affects the gut microbiota of seabream by increasing the abundance of beneficial genera, thereby improving gut health and maintaining growth performance of *S. aurata* from coastal farms.

**Supplementary Information:**

The online version contains supplementary material available at 10.1007/s10695-024-01314-9.

## Introduction

Aquaculture is one of the most efficient and sustainable methods of producing animal protein for human consumption. According to the Food and Agriculture Organization (FAO), aquaculture is expected to provide about 60% of seafood by 2030 (FAO 2020). However, to ensure sustainable and continued growth of this sector, the limit of feed scarcity must be surpassed. Marine ingredients traditionally used in aqua feeds, *i*.*e*., fishmeal (FM) and fish oil (FO), are limited resources with relatively stable production over the last decade. In addition, quotas and demand for human consumption constrain fisheries designated for the production of FM and FO in the European Union (EU).

Therefore, one of the major challenges of modern aquaculture is to find high-quality ingredients based on environmentally friendly resources as an alternative to FM and FO that can be used for feed formulations. The most promising new resources include low-trophic marine species (mesopelagic fish, zooplankton, polychaetes, macroalgae, and crustaceans), microbial ingredients (bacteria, yeast, and microalgae), insects (black soldier fly, yellow mealworm, and crickets), and animal by-products (poultry meal, blood meal, and hydrolyzed feather meal) (Albrektsen et al. 2022). Recently, insects have gained attention as an important sustainable nutrient source for animal feed, especially for fish (Barroso et al. 2014; Henry et al. 2015; Sogari et al. 2019; Alfiko et al. 2022). This is because insects are a natural food source for fish, especially carnivorous and omnivorous species, which are evolutionarily adapted to eat them as part of their regular diet (van Huis 2020).

To date, eight species have been approved by EU legislation (Regulation 2021/1925) for the production of feed in aquaculture, including silkworms (*Bombyx mori*), black soldier fly (*Hermetia illucens*), house fly (*Musca domestica*), yellow mealworm (*Tenebrio molitor*), lesser mealworm (*Alphitobius diaperinus*), house cricket (*Acheta domesticus*), banded cricket (*Gryllodes sigillatus*), and Jamaican field cricket (*Gryllus assimilis*). Among these, *H. illucens* (HI) has attracted the most interest from the aqua feed industry as an alternative protein source. HI larvae can efficiently grow on a variety of decomposing organic substrates and convert them into valuable nutrients (Meneguz et al. 2018; Bonelli et al. 2020; Hosseindoust et al. 2023). The HI larval meal produced from dried insect larvae contains high levels of protein (36–48% DM) and fat (31–33% DM), as well as essential amino acids (EAA), vitamins, and minerals (Oteri et al. 2021). Despite their undeniable nutritional qualities, the use of HIM in animal feed is still limited due to their imbalanced fatty acid profile (Barragan-Fonseca et al. 2017; Hoc et al. 2020). Indeed, larvae of HI are unable to synthesize polyunsaturated fatty acids (PUFAs) and are instead rich in saturated fatty acids (SFAs; mainly lauric, myristic, and palmitic) (Dalle Zotte et al. 2019; Hoc et al. 2020). Therefore, the main drawback is a lower content of omega-3 fatty acids, such as eicosapentaenoic acid (EPA) and docosahexaenoic acid (DHA), in final products derived from animals fed with larvae of these diptera. This is undeniably a problem for producers and consumers, especially in the seafood supply chain. Previous research has shown that it is possible to improve the fatty acid profile of HI larvae by enriching their rearing substrate with a source of n-3 PUFA, such as fish waste (Cullere et al. 2019). However, the modulation of the HI fatty acid profile by PUFA-enriched substrates has not always been successful, as recently reported by Ewald et al. (2020) and Ceccotti et al. (2022). Ewald et al. (2020) found that the fatty acid profile of larvae consisted mainly of lauric acid C12:0 and other SFAs endogenously synthesized by larvae, regardless of diet. In addition, the percentage of EPA and DHA tended to decrease with increasing weight, i.e., larvae with a higher weight contained a higher percentage of SFAs and a lower percentage of EPA and DHA. Furthermore, *H. illucens* larvae reared on substrates enriched in monounsaturated fatty acids (MUFAs) and PUFAs tended to bioaccumulate some of these fatty acids to promote the biosynthesis of lauric acid Hoc et al. (2020).

Currently, the potential of HI meal (HIM) as a valuable feed ingredient has been determined for several commercially important farmed fish species, including Atlantic salmon (*Salmo salar*), rainbow trout (*Oncorhynchus mykiss*), gilthead seabream (*Sparus aurata*), and European seabass (*Dicentrarchus labrax*) (Rimoldi et al. 2019, 2021; Terova et al. 2019; Bruni et al. 2020; Li et al. 2020; Biasato et al. 2022; Oteri et al. 2022; Anedda et al. 2023; Di Rosa et al. 2023; Zarantoniello et al. 2023). The overall results showed that the health status and production performance of the fish, as well as the quality of the products obtained from them, were satisfactory (Alfiko et al. 2022). Numerous studies on different aquaculture species have also demonstrated that replacing FM with HIM has a positive modulatory effect on gut bacterial communities and increases bacterial richness and abundance of beneficial lactic acid bacteria (Huyben et al. 2019; Rimoldi et al. 2019, 2021; Terova et al. 2019; Weththasinghe et al. 2022; Biasato et al. 2022; Hasan et al. 2023).

The HI contains bioactive compounds such as lauric acid and chitin that can affect the gut microbiota of fish. Lauric acid exerts potent antimicrobial activity against both Gram-positive and Gram-negative pathogens (Skřivanová et al. 2006; Kumar et al. 2020; Borrelli et al. 2021). In contrast, chitin, a biopolymer of glucosamine found in the exoskeletons of arthropods, has shown antimicrobial and bacteriostatic activity against several Gram-negative pathogens in addition to its prebiotic properties (Rangel et al. 2022a; Weththasinghe et al. 2022; Rimoldi et al. 2023). Chitin, which resembles cellulose, acts as an indigestible dietary fiber that promotes the growth of beneficial and chitin-degrading bacteria. Thus, we recently obtained promising results with the inclusion of insect exuviae, the main chitin-rich by-products of insect farming, in the diet of rainbow trout (Rimoldi et al. 2023). In particular, pupal exuviae improved the proportion of Firmicutes and Actinobacteria phyla in the gut of trout.

Although numerous publications have already described the use of insects as sustainable protein sources in fish feeds, most of these studies have been conducted on a small scale in a controlled environment, such as experimental facilities, to minimize variables. To date, there are few findings from large-scale trials. Accordingly, the present study investigated the effect of replacing FM with HIM in the diets of gilthead seabream (*S. aurata*) reared off shore. Growth performance, gut health, and gut microbiota profile were considered to monitor fish health and response to the diet. Two isolipidic and isoproteic diets were tested on farms: a control diet without insect meal and an experimental diet with 11% HIM as a replacement for FM. High-throughput 16S rRNA gene amplicon sequencing (MiSeq platform, Illumina) was used to characterize gut microbial communities.

## Materials and methods

### Ethics

The experiment was carried out at the “Maricolture Sarde Srl” farm located in Sant’Antioco, a region in South Sardinia, Italy. The Ethical Committee of the University of Messina, Department of Veterinary Sciences, granted approval for the experimental procedure (Authorization No. 082/2022).

### Diets

Two isolipid (approximately 18 g/100 g) and isoprotein (42 g/100 g) diets were manufactured by Veronesi S.p.A. (Verona, Italy). The diets meet the nutritional requirements of gilthead seabream. The extruded feeds were produced in a 4-mm-pellet size. The control basal diet (FM) contained only FM as a protein source of animal origin (250 g/kg), whereas the experimental diet (HIM) contained 11% defatted HIM (used as a basal diet) as a partial substitute for FM, which accounted for 35% of the substitute (Table 1).

**Table 1:** Raw materials and proximate composition of the diets

	FM	HIM
Ingredients (% as feed):		
Fishmeal	25.0	16.5
Wheat meal	18.0	14.5
Soybean meal	15.0	15.0
*Hermetia illucens* meal	-	11.0
Rapeseed oil	10.0	10.0
Sunflower meal	5.0	5.0
Fish oil	5.0	5.0
Wheat gluten	5.0	5.0
Corn gluten	5.0	5.0
SPC (soy protein concentrate)	5.0	5.0
Pea protein	4.0	4.0
Amino acids^a^, vitamin^b^, and mineral^c^ fraction	3.0	4.0
Proximate composition (% as feed):		
DM	95.05	94.71
CP	41.9	41.40
EE	19.16	18.98
Fiber	1.96	2.14
Ash	5.95	5.29
Gross energy (MJ/kg feed)	19.86	19.80

^a^See Tables 2 and 3

^b^Vitamin mixture (IU or mg per kg): vitamin A 12,000 IU; vitamin D3 2000 IU; vitamin E 160 mg; vitamin C (L-ascorbic acid) 160 mg

^c^See Tables 2 and 3

### Chemical analysis of feeds

All the chemical analyses of the feeds were performed in triplicate.

Dry matter (DM, AOAC #930.15), crude protein (CP, AOAC #2001.11), crude fiber (AOAC #978.10), ash (AOAC #942.05) contents, and ether extract (EE, AOAC #920.39) were determined according to the standard procedures of Association of Official Analytical Chemists (AOAC 2019). Gross energy (GE) was evaluated using an adiabatic calorimetric bomb. The proximate composition of the diets is reported in Table 1. As regards the composition of fatty acids, amino acids, and minerals of the insect meal and the two diets, the analytical methods were described in detail by Oteri et al. (2021), and the results are reported in Tables 2, 3 and 4

**Table 2:** Fatty acid composition (g/100 g dry matter) of two experimental diets

Item	FM	HIM
C10:0	0.01	0.08
C12:0	0.06	2.81
C13:0	0.02	0.02
C14:0	2.08	2.53
C15:0	0.29	0.26
C16:0	11.53	11.73
C16:1	3.08	3.03
C17:0	0.33	0.26
C18:0	3.42	3.47
C18:1n9	47.08	45.01
C18:1n7	0.14	0.17
C18:2n6	14.81	14.65
C18:3n6	0.06	0.06
C18:3n3	5.29	5.03
C20:0	0.49	0.48
C20:1n9	1.12	1.05
C20:2n6	0.13	0.12
C20:3n3	0.47	0.44
C20:4n6	0.05	0.04
C20:5n3	4.14	3.79
C22:0	0.30	0.28
C22:1n9	0.23	0.22
C22:2n6	0.03	0.02
C23:0	0.03	0.03
C24:0	0.55	0.54
C22:6n3	4.26	3.91
SFA	19.11	22.48
MUFA	51.66	49.48
PUFA	29.23	28.04
SFA/UFA	0.24	0.29
n3-PUFA	14.16	13.16
n6-PUFA	15.08	14.88
EPA/DHA	8.39	7.69

*SFA* saturated fatty acids, *MUFA* monounsaturated fatty acids, *PUFA* polyunsaturated fatty acids, *SFA/UFA* saturated fatty acids/unsaturated fatty acids ratio, *EPA/DHA* eicosapentaenoic acid/docosahexaenoic acid ratio

**Table 3:** Amino acid composition (g/100 g dry matter) of the experimental diets

	FM	HIM
Essential amino acids (EAA)		
Arginine	2.29	2.81
Histidine	1.37	1.29
Isoleucine	2.32	2.13
Leucine	4.51	4.40
Lysine	4.34	4.65
Hydroxylysine	0.79	0.57
Methionine	0.63	0.63
Phenylalanine	2.87	3.29
Threonine	1.54	1.49
Valine	2.33	2.05
Tryptophan	0.17	0.15
Non-essential amino acids (NEAA)		
Alanine	2.30	2.14
Aspartic acid	3.94	3.69
Cysteine	0.15	0.20
Glycine	1.71	1.64
Glutamic acid	2.90	3.15
Proline	2.41	2.18
Hydroxyproline	0.90	0.89
Tyrosine	1.77	1.56
Serine9	1.88	2.07
EAA/NEAA	1.29	1.34

**Table 4:** Mineral element profile (mg/kg dry matter) of the experimental diets

Element	FM	HIM
Zn	126	130
Fe	128	148
Ba	0.5	2.1
Ti	< 1	< 1
Ag	< 1	< 1
Hg	< 1	< 1
Mn	64	85
Al	194	251
Cu	16	16
Pb	< 1	< 1
As	< 1	< 1
Cd	< 1	< 1
Cr	3.9	6.5
Ni	< 1	< 1
**Se**	** < 1**	** < 1**

### Fish rearing conditions and sampling

The feeding experiment started on November 2, 2021, and the 25-week feeding experiment was carried out at the Maricolture Sarde Srl fish farm (Sant’Antioco, Sardinia, Italy). Approximately 60,000 gilthead sea bream (average initial body weight 131 ± 1.4 g) were randomly distributed to four square coastal cages (12 × 12 × 1.5 m) with an initial biomass of approximately 2000 kg per cage. During the experimental period, fish were hand-fed either FM or HIM at a daily meal (between 08:00 and 10:00), 6 days a week (two cages/diet). Feeding rates ranged from 0.6 to 1.3% of biomass, depending on water temperature. Biomass in each cage was determined by weighing 100 fish in bulk each month. Fish mortality in the cages was monitored at 3-week intervals. After 25 weeks experimentation, 88 fish per diet were sacrificed for measurement of growth parameters. Specifically, individual final body weight was determined for all fish (*n* = 88 per diet) to calculate the following indices for each diet group:

$$\begin{array}{c}\mathrm{WG}\;(\mathrm{Weight}\;\mathrm{gain},\;\mathrm g)=\;\mathrm{Final}\;\mathrm{body}\;\mathrm{weight}(\text{g})-\mathrm{Initial}\;\mathrm{body}\;\mathrm{weight}(\text{g})\\\mathrm{FCR}\;(\mathrm{Feed}\;\mathrm{conversion}\;\mathrm{rate})=\;\mathrm{Total}\;\mathrm{feed}\;\mathrm{consumed}\;\mathrm{per}\;\mathrm{cage}\;(\mathrm g\;\mathrm{DM})/\;\mathrm{Weight}\;\mathrm{gain}\;(\text{g})\\\text{SGR}\left(\mathrm{Specific}\;\mathrm{growth}\;\mathrm{rate},\%/\text{day}\right)\;=\;\left(\text{In}\left(\mathrm{final}\;\mathrm{body}\;\mathrm{weight},\text{g}\right)-\text{In}\left(\mathrm{initial}\;\mathrm{body}\;\mathrm{weight},\text{g}\right)\right)/\mathrm{Number}\;\mathrm{of}\;\mathrm{feeding}\;\mathrm{days}\rbrack\times\;100.\end{array}$$

From a subsample of 24 specimens (12 fish per diet, 6 fish per cage), the liver was separated and its weight measured to calculate the hepatosomatic index (HSI) for each fish as follows:

$$\mathrm{HSI\;}(\mathrm{\%})\;=\;(\mathrm{liver\;weight }({\text{g}})/\mathrm{body\;weight\;}({\text{g}}))\;\times\;100$$

Samples of the liver and the intestine (divided into proximal and distal parts) were collected from ten fish per diet and fixed in a solution of neutral buffered formalin (NBF, 10%) to perform histological analysis.

Similarly, for gut microbiota analysis, 10 fish per diet were sacrificed and the whole intestine (without pyloric ceca) was collected from each fish aseptically. The digesta- and mucosal microbiota were collected and mixed together as described in detail by Rimoldi et al. (2020).

Finally, nine fish were sampled per tank and three fecal samples were collected from each tank for volatile fatty acid analysis. All the fish were killed by putting them in ice water.

### Histology

NBF-fixed liver and intestinal specimens were embedded in paraffin and cut into 5-m slices with a microtome (Leica RM2245). The acquired slides were stained with hematoxylin and eosin (H&E), and the tissue morphology was studied using a CMOS Discovery C30 digital camera under a light microscope (Zeiss Axiophot microscope). Fiji software (an open-source Java-based imaging package) was used to process the captured images. The following intestinal morphological characteristics were examined, as specified by Escaffre et al (2007): villus height (ViH), villus width (ViW), and submucosal layer thickness (SMT). A semi-quantitative grading approach based on a grading score (1 = not observed/low, 2 = moderate, 3 = severe) was employed for the liver (Caballero et al. 2004; Rodrigues et al. 2017; Camargo and Martinez 2007). In particular, vacuolization of hepatocytes (HV), nuclear displacement (ND), cellular hypertrophy (CH), and irregular nuclear shapes (NS) were taken into consideration as histological characteristics in the liver.

### Taxonomic and functional characterization of gut microbiota

Bacterial genomic DNA isolation, amplicon library construction, and sequencing.

Bacterial DNA was extracted from each sample (feces + mucosa) and from three aliquots of 200 mg per feed. The DNeasy® PowerSoil® Pro kit (Qiagen, Italy) was used for extraction according to the manufacturer’s instructions, with an additional mechanical lysis step as previously described (Rimoldi et al. 2019). A negative control, with lysis buffer only, was processed in parallel with the biological samples to exclude any external contamination. The extracted DNA was quantified spectrophotometrically and then stored at − 20 °C until use. The 16S amplicon sequencing library was prepared by the microbiome sequencing service GALSEQ srl (Milan, Italy) and sequenced on an Illumina MiSeq sequencing platform. All steps of preparation and sequencing of the 16S rRNA gene library have been described previously (Terova et al. 2019). Briefly, the V4 hypervariable region of the 16S rRNA gene was amplified with oligonucleotides 515F: 5′-GTGYCAGCMGCCGCGGTAA-3′ and 806R: 5′-GGACTACNVGGTWTCTAAT-3′. Each library was diluted to 5 ng/µL, mixed at equimolar concentration (4 nM), and then subjected to multiplex sequencing on an Illumina MiSeq instrument using a 2 × 150-bp PE strategy.

### DNA sequencing data analysis

Raw next generation sequencing data were analyzed using the QIIME2TM pipeline (v. 2020.2) (Bolyen et al. 2019). The entire applied QIIME bioinformatics workflow was previously described by Terova et al. (2021). The final result was an amplicon sequence variant (ASV) table with high-quality filtered reads. Taxonomy was assigned to ASVs using the reference database SILVA (https://www.arb-silva.de/) (Quast et al. 2013). ASVs assigned to chloroplasts and mitochondria were removed from the analysis. The qiime 2 core-metrics-phylogenetic pipeline was applied to calculate alpha and beta diversity. Alpha diversity was assessed at a depth of 31,081 reads, and both species richness (Chao 1, observed ASV) and biodiversity (Faith PD, Shannon and Simpson) were calculated. The weighted and unweighted UniFrac distance matrices (Lozupone and Knight 2005; Lozupone et al. 2007) were used to estimate beta diversity.

Only ASVs present in at least 60% of the samples in each group were considered in the analysis of core microbiota.

The free tool Venny 2.1 (https://bioinfogp.cnb.csic.es/tools/venny/index.html) was used to create a Venn diagram depicting unique and common ASVs between different feeding groups.

### Bacterial metabolic pathway prediction

The functional profile of gut microbial communities was predicted using the PICRUSt (Phylogenetic Investigation of Communities by Reconstruction of Unobserved States) tool (Langille et al. 2013) based on the KEGG (Kyoto Encyclopedia of Genes and Genomes) database. Differences between two experimental groups (FM and HIM) were validated with a two-sided Welch *t*-test using the Statistical Analysis of Metagenomics Profiles software package (STAMP, v.2.1.3) (http://kiwi.cs.dal.ca/Software/ STAMP) (Parks et al. 2014).

### Analysis of fecal short-chain fatty acids (SCFAs)

Qualitative and quantitative determinations of SCFAs (acetate, propionate, iso-butyrate, and butyrate) were performed according to the modified extraction method of Chlebicz-Wójcik and Śliżewska (2020). For this purpose, 0.5 g of fecal matter was placed in a falcon, dissolved with 5 mL of water for HPLC PLUS, and vortexed for 3 min. Immediately thereafter, the samples were centrifuged at 4000 rpm for 30 min. The supernatant was filtered through a 0.22-µm PTFE syringe filter. The extracts obtained were analyzed using a HPLC–UV-VIS system (Shimadzu, Milan, Italy) equipped with two LC -20AD pumps, a CBM-Alite controller, a DGU-20A5 degasser, and an automatic injector.

Chromatographic separation was performed using a 150-mm × 4.6-mm I.D. and 2.7-µm particle Ascentis Express 90A C18 column (Merck KGaA, Darmstadt, Germany) in isocratic mode. The mobile phase consisted of 0.005 M H_2_SO_4_ with a flow rate of 0.6 mL/min and a column temperature of 60 °C. The injection volume was 10 µL. The wavelength for UV–VIS analysis was set to 210 nm. The software used for data processing is LC solution (Shimadzu, Milan, Italy). Qualitative analyses of SCFAs were performed by comparing the retention times of each certified standard, whereas quantitative evaluations were performed using the calibration curve method in a range of different concentrations. The results were expressed in millimoles per liter.

### Statistics

The Shapiro–Wilk test was employed to assess normality, while homoscedasticity was evaluated using Levene’s test. The application of angular transformation was employed in order to linearize sigmoid distributions and account for variations in the relative frequencies of bacterial species. The statistical tests employed to compare growth performances, alpha diversity indices, and bacterial abundances of the two experimental groups were the Student’s *t*-test or the Mann–Whitney *U*-test, depending on whether the data exhibited a normal distribution. The statistical technique employed in this study to assess variations in beta diversity among different groups was permutational multivariate analysis of variance (PERMANOVA). The statistical analyses were conducted using Past4 v. 4.02 software (Hammer et al. 2001), with a significance level of *p* < 0.05.

## Results

### Growth performance

During the experiment, both diets were well accepted by the fish. Regardless of the feed, a mortality rate of 10% was observed. The results on growth performance of gilthead seabream fed the experimental diets are shown in Table 5. Feeding HIM had no significant effect on growth or somatic index. At the end of the 25-week feeding trial, final body weight (HIM 244 g vs. FM 246, *p* = 0.707), specific growth rate (HIM 0.21% vs. FM 0.22%, *p* = 0.720), feed conversion ratio (HIM 1.32 vs. FM 1.44, *p* = 0.138), and hepato-somatic index (HIM 1.20% vs. FM 1.15%, *p* = 0.512) did not differ between the two feeding groups (Table 5).

**Table 5:** Growth performance and hepatic somatic index (HSI) of *S. aurata* fed two experimental diets. SGR, specific growth rate; BW_f_, final body weight; FCR, feed conversion ratio

	BW_f_	SGR	FCR	HSI
FM	246 ± 40	0.22 ± 0.09	1.44 ± 0.43	1.15 ± 0.21
HIM	244 ± 36	0.21 ± 0.09	1.32 ± 0.54	1.20 ± 0.17
*p*-value	0.707	0.720	0.138	0.512

### Gut and liver morphology

**Fig. 1:**
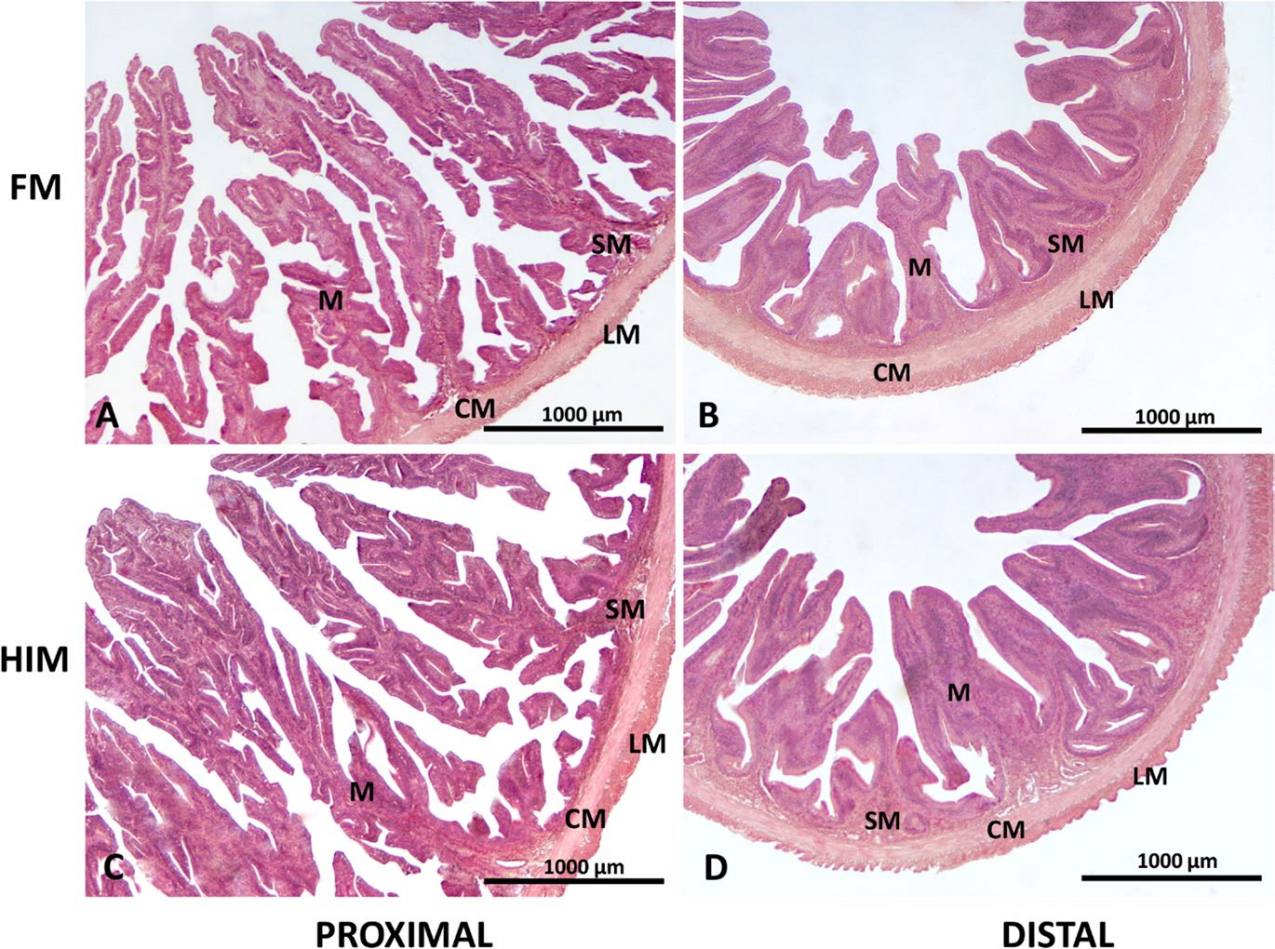
Standard hematoxylin and eosin (H&E) staining of proximal and distal intestinal sections of FM (panels **A**, **B**) and HIM (panels **C**, **D**). M, mucosa; SM, submucosa; CM, circular muscle layer; LM, longitudinal muscle layer. Scale bar, 500 μm

**Fig. 2:**
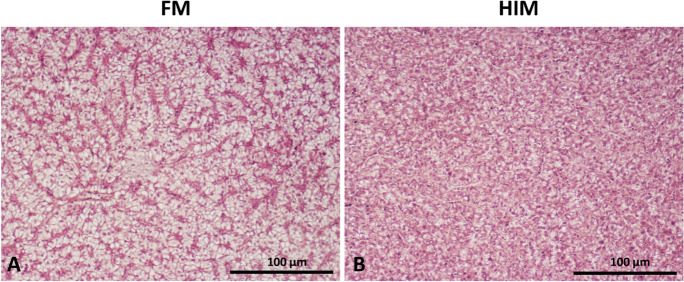
Standard hematoxylin and eosin (H&E) staining of liver from FM (**A**) and HIM fish (**B**). Scale bar, 500 µm

The gross morphology of the gastrointestinal system and liver exhibited preservation, without any apparent indications of inflammation (Figs. 1 and 2). The tissue structure of both the proximal and distal regions of the intestine exhibited a well-organized and well-preserved state, devoid of any apparent indications of injury or inflammation, irrespective of the dietary conditions (see Fig. 1). The mucosal folds in the proximal intestine exhibited a higher degree of organization and a greater abundance compared to those in the distal intestine (see Fig. 1A, C). Although the observed trend in muscle layer thickness and villus height in both the proximal and distal intestines of fish fed the HIM diet was not found to be statistically significant, it is worth noting (Fig. 1; Table 6).

**Table 6:** Morphometry of proximal and distal intestinal segments of *S. aurata* fed two experimental diets (*n* = 10)

	FM	HIM	*p*-value
Proximal intestine			
Villus height (μm)	866.40 ± 63.39	1033.07 ± 63.59	0.08
Muscle thickness (μm)	45.12 ± 1.26	51.27 ± 4.29	0.19
Villus width (μm)	136.13 ± 4.06	139 ± 5.20	0.67
Distal intestine			
Villus height (μm)	645.60 ± 31.81	722.64 ± 24.85	0.06
Muscle thickness (μm)	82.75 ± 1.74	86.43 ± 1.65	0.13
Villus width (μm)	109.62 ± 4.85	113.39 ± 3.38	0.52

The preservation of the liver parenchyma was observed to be satisfactory in both groups of fish, similar to the condition observed in the gut. The liver in all dietary groups displayed a typical parenchymatic architecture, which was characterized by a consistent arrangement of hepatic cords separated by sinusoids and bile ducts (Fig. 2). The lipid infiltration observed in the fish from FM was of moderate intensity, leading to the relocation of the nuclei towards the outer region of the hepatocytes, as depicted in Fig. 2A. The histological assessment, as presented in Table 7, indicated the absence of apparent indications of structural impairment or inflammation resulting from the consumption of the insect-based diet.

**Table 7:** Results of histological scoring of livers of gilthead seabream fed two different experimental diets (*n* = 10)

	FM	HIM	*p*-value
Nuclear displacement (ND)	1.27	1.14	0.35
Cellular hypertrophy (CH)	1.27	1.16	0.52
Irregular nuclei shapes (NS)	1.18	1.04	0.24
Hepatocytes vacuolization (HV)	1.27	1.22	0.76

Grading scale: 1 = not observed/minimal, 2 = moderate, 3 = severe

### Illumina sequencing efficiency

The sequencing process was conducted with remarkable efficiency, resulting in a total of 1,082,214 high-quality sequences. Among these sequences, 910,820 were from intestinal samples, while the remaining 171,394 were derived from feed pellets. A mean Good’s coverage value over 99% was achieved, suggesting that the dataset adequately represents the bacterial communities. The sequencing depth for computing alpha diversity indices was determined to be 31,081 reads based on the analysis of rarefaction curves. The raw sequencing data were deposited in the public database of the European Nucleotide Archive (EBI ENA) with the accession code PRJEB64777.

### Profiles of feed-associated microbial communities

**Fig. 3:**
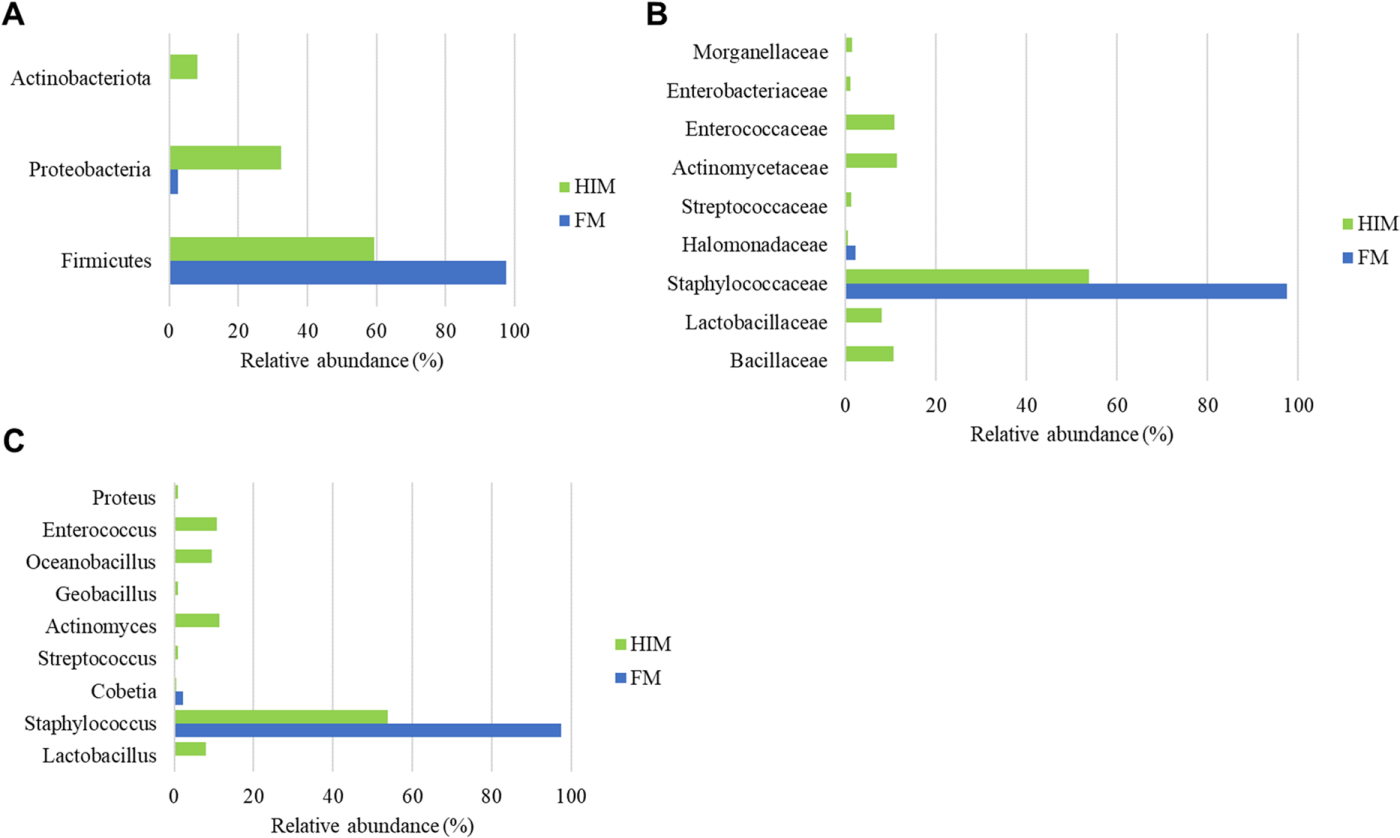
Profiles of the feed-associated microbiota at the taxonomic level of phylum (**A**), families (**B**), and genera (**C**). Only taxa with a total abundance of ≥ 0.5% are reported. Bacteria with lower abundance are grouped together and referred to as “others”

The utilization of 16S rDNA gene amplicon sequencing metabarcoding analysis was employed to examine the feed-associated microbiota, leading to the identification of noteworthy distinctions between two distinct feeds. The microbial compositions of the feeds were analyzed, focusing on the most prevalent taxa (≥ 1% at the order level and ≥ 0.5% at the family and genus levels). The analysis revealed the presence of three phyla, three classes, six orders, nine families, and nine genera (Fig. 3A–C). The comprehensive compilation of bacterial taxa categorized by genera is included in Supplementary Data File S1. The results of the alpha diversity analysis revealed that the microbial community linked to the HIM feed had the greatest levels of species richness, as shown by the Chao1 and observed ASV metrics, as well as biodiversity, as measured by the Shannon, Simpson, and Faith-PD indices (Table 8).

**Table 8:** Alpha diversity indexes of feed-associated microbiota (*n* = 3) and gut microbial communities (*n* = 10). Different letters indicate differences between means (*p* < 0.05)

	Chao1	Obs ASVs	Faith_PD	Shannon	Simpson
Feed samples					
FM	120 ± 19^b^	109 ± 11^b^	1.51 ± 0.07^b^	3.71 ± 0.04^b^	0.84 ± 0.002^b^
HIM	390 ± 19^a^	381 ± 23^a^	3.58 ± 0.10^a^	6.10 ± 0.27^a^	0.95 ± 0.005^a^
Gut samples					
FM	244 ± 55	227 ± 50	2.44 ± 0.93	4.95 ± 0.46	0.92 ± 0.02
HIM	267 ± 70	249 ± 68	2.90 ± 0.85	4.70 ± 0.61	0.89 ± 0.03

Consistent with the alpha diversity analysis, a significant decrease in taxa was observed in the FM feed. At the phylum level, the microbiota in the FM feed consisted mainly of Firmicutes (97.6%), with the remainder consisting of Proteobacteria (2.4%) (Fig. 3A). In contrast, diets containing insect meal had a higher proportion of Proteobacteria (32%) and a lower abundance of Firmicutes (60%) compared to FM. In addition, the Actinobacteriota phylum (8%) was found only in association with HIM feeds (Fig. 3A). Bacteria belonging to the families Actinomycetaceae (11.3%), Enterococcaceae (10.8%), Lactobacillaceae (7.8%), Morganellaceae (1.4%), Streptococcaceae (1.2%), and Enterobacteriaceae (1.1%) were detected only in the feed HIM. The family Staphylococcaceae (97.5%) was the major taxa in the control feed FM, followed by Halomonadaceae (2%) (Fig. 3B). In contrast, the Bacillaceae family was the most abundant in the HIM feed (10%). Accordingly, at the genus level, the genera *Actinomyces* (11.3%), *Enterococcus* (10.8%), *Oceanobacillus* (9.4%), *Lactobacillus* (7.8%), *Proteus* (0.97%) *Streptococcus* (0.96%), and *Geobacillus* (0.95%) were only detectable in the HIM feed. In contrast, the control feed FM showed an association with the genera *Staphylococcus* and *Cobetia* (Fig. 1C).

### Profiles of gut microbial communities and their dietary modulation

**Fig. 4:**
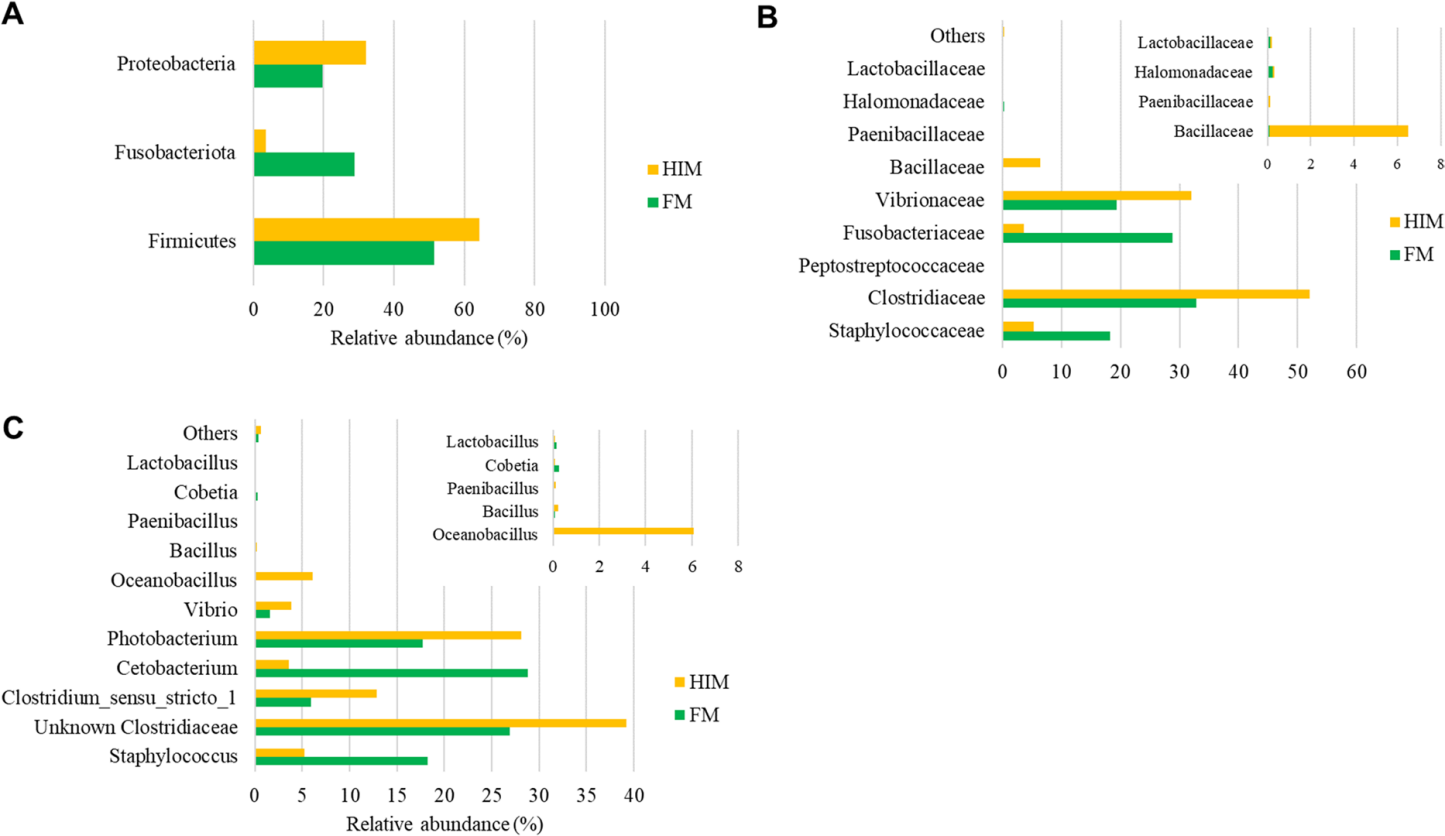
Profiles of the gut microbiota of gilthead sea bream fed two tested diets. The relative abundance of the overall most abundant bacterial phyla (**A**), families (**B**), and genera (**C**) is indicated. Bacterial taxa with lower abundance (< 0.5%) are summarized and labelled as “others”

Irrespective of dietary variations, a comprehensive characterization of the microbiota composition in all intestinal samples was achieved, providing detailed information at the genus level. This analysis revealed the presence of 6 phyla, 8 classes, 18 orders, 22 families, and 33 genera, as documented in Supplementary Data File S2. When considering solely the most representative taxa, the assemblage was composed of three phyla, four classes, five orders, nine families, and ten genera. The gut microbiota profiles of two distinct feeding groups are depicted in Fig. 4A–C, showcasing the taxonomic composition at the phylum, family, and genus levels.

**Fig. 5:**
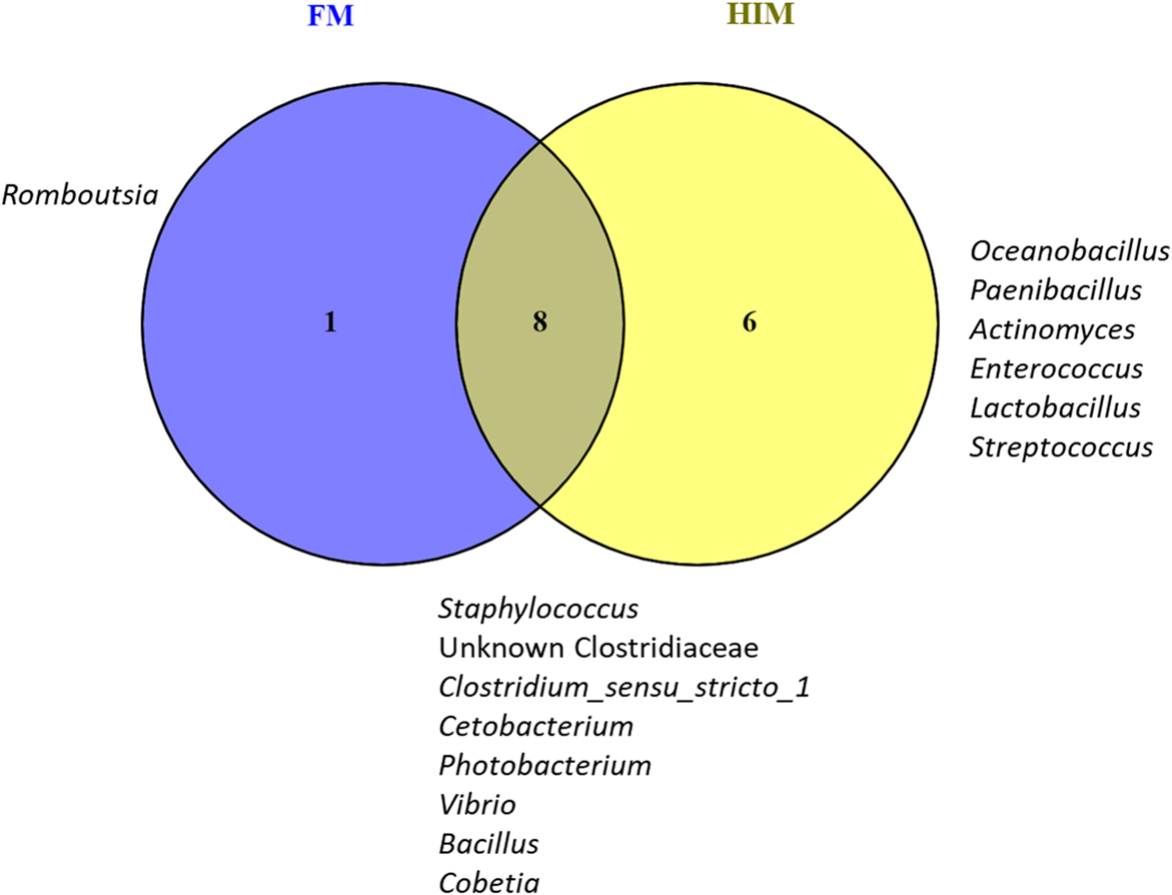
Core microbiota. Venn diagram showing unique and common taxa between two experimental groups

**Fig. 6:**
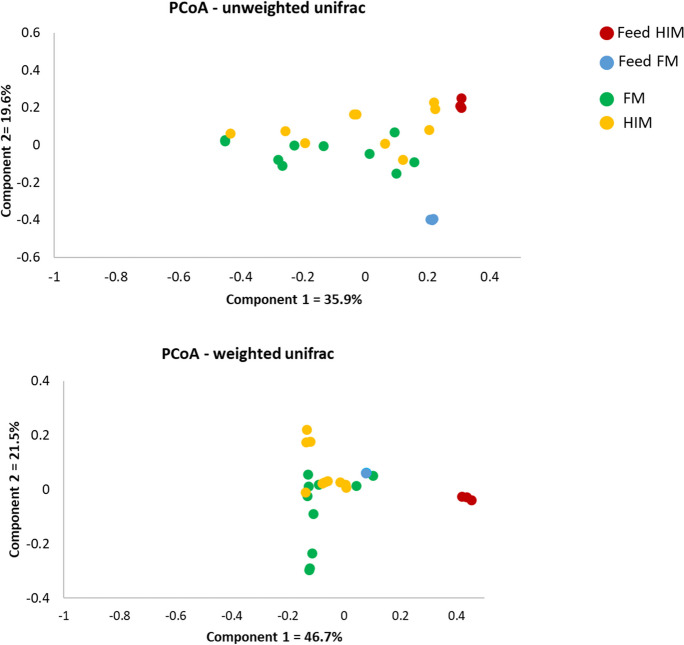
Principal coordinate analysis (PCoA) of unweighted and weighted UniFrac distances of gut microbial communities. The plots show the 2D representation of each fish and feed sample according to their microbial profile at the genus level

The examination of alpha diversity indicated that there were no significant differences observed between the two feeding groups, as shown in Table 5. According to the Venn diagram, it is evident that the common core microbiota consisted of only eight species. Moreover, the HIM fish were found to have six genera solely assigned to them, whereas the FM fish had one genus exclusively assigned to them (refer to Fig. 5). The use of multivariate permutational analysis, utilizing both weighted and unweighted UniFrac metrics, demonstrated statistically significant distinctions (*p* < 0.05) in the gut bacterial communities of the two feeding groups. Additionally, notable dissimilarities were observed between the gut microbiota and the diet microbiota, as indicated in Table 9. Consequently, the use of principal coordinate analysis (PCoA) on the unweighted and weighted UniFrac metrics resulted in distinct clusters for gut and feed samples, with gut samples further grouped based on diet (Fig. 6).

**Table 9:** Results of PERMANOVA analysis on unweighted and weighted UniFrac metrics data

Group 1	Group 2	Size	Permutations	Pseudo-*F*	*p*-value
Unweighted UniFrac					
FM	HIM	20	999	2.224	0.043
FM	Feed FM	13	999	5.616	0.003
HIM	Feed HIM	13	999	3.688	0.015
Feed FM	Feed HIM	6	999	75.116	0.094
Weighted UniFrac					
FM	HIM	20	999	3.653	0.021
FM	Feed FM	13	999	4.546	0.007
HIM	Feed HIM	13	999	24.349	0.004
Feed FM	Feed HIM	6	999	355.807	0.094

**Fig. 7:**
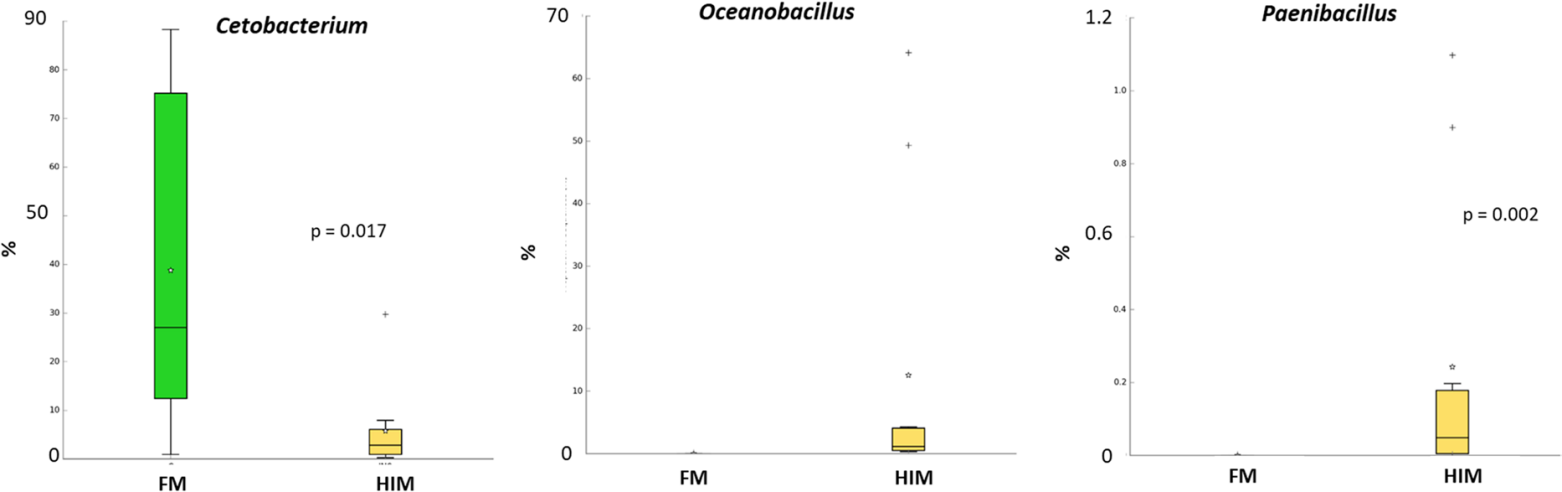
Bacterial genera with significant influence of diet. Welch’s *t*-test (*I* < 0.05)

At the taxonomic level of phylum, the three most prevalent phyla identified were Firmicutes, Proteobacteria, and Fusobacteria (Fig. 4A). The dietary composition had an impact on Fusobacteria, as evidenced by the increased proportion of Fusobacteria in the group of fish that were fed the control diet. In accordance with the findings, the bacterial family Fusobacteriaceae exhibited a higher prevalence in the FM group, but Bacillaceae and Paenibacillaceae have shown increased abundance in the gastrointestinal tract of fish that were given an insect-based diet (Fig. 4B). At the level of genus, the statistical analysis using Welch’s *t*-test indicated that the diet had a significant impact on only three genera (*p* < 0.05) as shown in Fig. 7. The group of fish designated as the control exhibited a greater relative abundance of *Cetobacterium*, but the species *Oceanobacillus* and *Paenibacillus* were mostly discovered exclusively in the fish group HIM (Fig. 7).

### Microbial metabolic pathway prediction

**Fig. 8:**
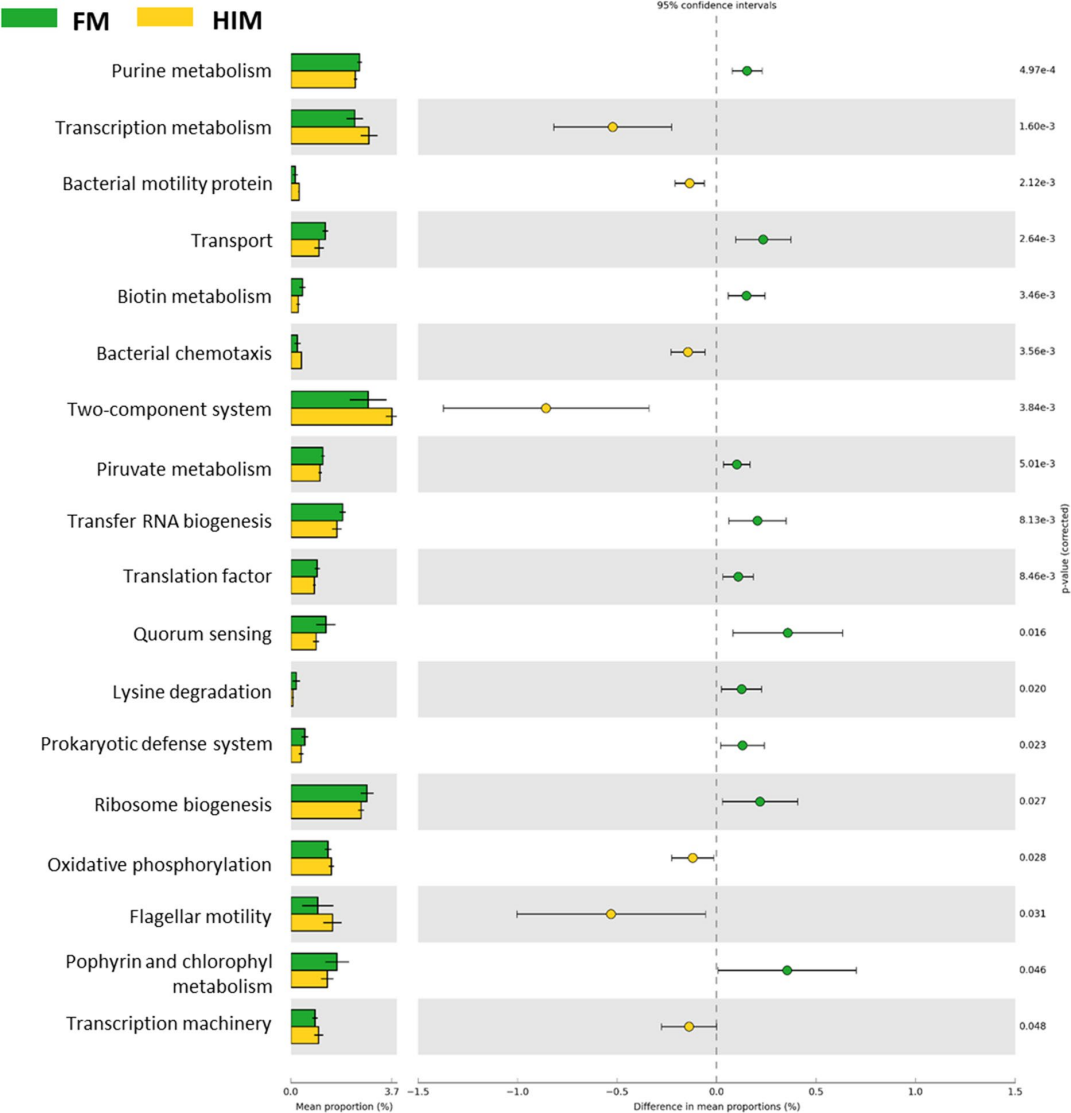
Results of PICRUSt analysis of predicted functional pathways in gut microbiota of control (FM) and HIM fish groups. Extended error bar graph and statistical analysis were generated using the bioinformatics software STAMP

The metabolic activities of microbial communities were estimated using the PICRUSt software, which utilizes a comparative analysis of discovered 16S rRNA gene sequences with those of known genome-sequenced taxa to infer the potential gene contents of microbial communities. A statistically significant difference was seen in a total of 18 pathways between the two feeding groups, as depicted in Fig. 8. It is noteworthy that fish that were provided with an insect-based diet had an elevated expression of genes associated with bacterial chemotaxis, motility, and the two-component signal transduction system.

### Volatile SCFAs in fecal samples

The consumption of insects in the HIM group led to a noteworthy rise in short-chain fatty acids (SCFAs), specifically acetic and propionic acids, in comparison to the FM diet (*p* < 0.05). In contrast, there were no significant differences seen in the amounts of butyric acid and isobutyric acid in fecal samples between the two experimental groups, as indicated in Table 10.

**Table 10:** Volatile SCFA content in fecal samples of two experimental groups. Results are expressed in millimoles per liter, mean ± SD (*N* = 12). *p < 0.05; ***p < 0.001; ns, *p* > 0.05

	Acetate (C2:0)	Propionate (C3:0)	Butyrate (C4:0)	Isobutyrate (C4:0)
FM	22.85 ± 2.00	7.52 ± 0.81	1.72 ± 0.16	0.44 ± 0.24
HIM	24.40 ± 1.43	9.90 ± 0.74	1.70 ± 0.11	0.66 ± 0.10
*Sig*	*	***	ns	ns

## Discussion

To our knowledge, this is the first farm-scale study to investigate the change in gut microbiota and production of SCFAs following the administration of insect larvae meal in gilthead seabream. Several studies, especially in salmonids, investigated the effects of HI on the gut microbial profile. However, the results were usually obtained in fish reared in indoor or outdoor tanks under controlled environmental conditions, and few of these studies focused on seabream (Fabrikov et al. 2021; Naya-Català et al. 2021; Panteli et al. 2021; Piazzon et al. 2022; Busti et al. 2024).

During the 25-week feeding trial, the experimental diet HIM, which contained 11% insect meal, was well accepted by the fish and did not significantly affect growth performance. Accordingly, it has been recently reported that FM can be replaced (up to 35%) by a moderate admixture of HI meal without negative effects on growth parameters or gut histological characteristics of seabream (Carvalho et al. 2023; Di Rosa et al. 2023). In contrast, gilthead seabream showed lower growth and higher (poorer) feed conversion when fed diets with a low FM content, in which 10% insect meal was equivalent to 66% of the FM replacement (Carvalho et al. 2023). This indicates that the gilthead sea bream is very sensitive to the chitin content in a modern diet with a low FM content (5%) (Carvalho et al. 2023).

Chitin is present in insect meals as it is the main component of the insect exoskeleton. As it is not clear which fish species can digest chitin, it is generally assumed that it could negatively affect the nutrient digestibility of carnivorous fish (Gasco et al. 2019). In fact, it appears that fish tolerance to chitin initially depends on the fish species. For example, studies on Atlantic salmon and rainbow trout showed that a complete replacement of FM with insect meal in practical feeding is possible without negatively affecting growth, while simultaneously increasing FCR (Lock et al. 2016; Belghit et al. 2019; Chemello et al. 2020). For gilthead seabream, Carvalho and colleagues (2023) set a tolerance of 0.7% (0.7 g/kg) chitin. Unfortunately, the chitin content in our HIM feed was not measured to confirm this. Furthermore, the optimal level of insect meal seems to depend on the FM content in the feed. In the current study, the HIM diet had a relatively high FM content (16.5%), which was sufficient to meet the total nutritional requirements of the seabream and prevent not only growth losses but also morphometric and histopathological changes in the intestinal tract.

In agreement with our results, HIM was added up to 15% without negative effects on growth performance and feed conversion in gilthead seabream when FM was kept at a minimum of 10% (Busti et al. 2024). The amount of FM was successfully reduced to as low as 5%, even with a 10% proportion of HIM, when processed animal proteins from poultry were used in combination with FM in the diet of gilthead seabream (Bosi et al. 2021). Higher levels of HIM were achieved in gilthead seabream fed diets containing between 25 and 45% FM, a level that is no longer considered sustainable for aquafeed formulation (Panteli et al. 2021; Mastoraki et al. 2022; Karapanagiotidis et al. 2023).

Histological evaluation of the intestinal morphology did not reveal any changes in the mucosa or signs of inflammation. In agreement with our results, no abnormalities in gross intestinal morphology and no obvious signs of inflammation were observed in gilthead seabream fed a diet containing 10% HIM (Bosi et al. 2021).

To further confirm this, in another pilot experiment conducted in marine RAS, changes in villus morphology were observed only in the anterior intestine of seabream fed a diet in which 50% of FM was replaced with HI meal, corresponding to a proportion of about 16% of insect meal inclusion, but not in a 35% FM replacement with an 11% proportion of insect meal inclusion (Di Rosa et al. 2023).

Like gut, liver health assessment is also an important indicator of nutritional status and general well-being of fish (Rašković et al. 2011). The HSI showed similar values in fish fed HIM compared to control fish, confirming that there were no problems associated with insect meal ingestion and indicating an adequate supply of taurine or its precursors cysteine and methionine in the HIM diet, as reported by other studies which tested similar diet formulations (Oteri et al. 2021). Taurine is a nonessential metabolite involved in the regulation of lipid metabolism and facilitates digestion and absorption of dietary lipids in the intestine (Ripps and Shen 2012). For example, in Atlantic salmon, partial substitution of FM with high levels of inclusion of insect meal (60%) resulted in negative changes in viscerosomatic index and HSI, and these changes correlated with low dietary taurine content (Lock et al. 2016; Belghit et al. 2018). In the present study, inclusion of insect meal in the diet did not result in significant changes or signs of inflammation in hepatic tissue. Furthermore, histological analysis of the liver revealed no significant differences in lipid accumulation between the two feeding groups. The histomorphological features observed in the livers of our fish are consistent with results previously reported in gilthead seabream (Randazzo et al. 2021; Anedda el al. 2023; Di Rosa et al. 2023;) and in other fish species, such as rainbow trout (Biasato et al. 2022; Palomba et al. 2022), Atlantic salmon (Belghit et al. 2019), and sea bass (Zarantoniello et al. 2023). In contrast, in sea bass, the highest FM replacement (20%) with full-fat spirulina-enriched HIM resulted in increased fat accumulation in the liver compared to control fish fed conventional marine diets (Zarantoniello et al. 2023). This could be due to the different ratio of n-6/n-3 PUFAs in the diets, which may lead to a high n-6/n-3 ratio and thus impair lipid metabolism and promote hepatic fat accumulation in fish (Ferramosca and Zara 2014; Dong et al. 2023). In the present study, the n-6/n-3 ratio was similar in the two diets tested and the HIM diet included a defatted HI meal, which likely helped prevent hepatic steatosis or hepatocellular disease.

Nowadays, insect meal products are considered a promising alternative for FM, not only as a valuable source of proteins and lipids, but also as a functional ingredient that strengthens the immune system and protects against pathogens (Somayeh et al. 2020). These health-promoting effects are largely attributed either to the direct broad-spectrum activity of the antimicrobial peptides produced by most insects (Sahoo et al. 2021) or to the indirect release of chitin or other bioactive compounds, such as medium-chain fatty acids. In particular, HI is characterized by a high content of lauric acid, which has been shown to have an inhibitory effect on several Gram-positive bacteria, such as *Clostridium perfringens*, *Staphylococcus aureus*, and D-*Streptococci* (Skrivanova et al. 2006; Spranghers et al. 2018). Indeed, dietary intake of a specific mixture of 1-monoglycerides of short and medium fatty acids (including lauric acid) positively modulated the gut microbiota of seabream by increasing beneficial lactic acid bacteria and reducing potentially pathogenic gamma proteobacteria (Rimoldi et al. 2018). Similarly, the indigestible polysaccharide chitin acts as a prebiotic that stimulates the growth of beneficial bacteria (Rangel et al. 2022a; Rimoldi et al. 2023) and has a bacteriostatic effect against various harmful Gram-negative bacteria (Nawaz et al. 2018).

In the last decade, several studies have shown that feeds enriched with insect meal are able to alter the microbial communities in the fish gut by increasing the abundance of the families Bacillaceae, Lactobacillaceae, and Actinobacteria (Huyben et al. 2019; Rimoldi et al. 2021, 2019; Terova et al. 2021, 2019; Bruni et al. 2020; Li et al. 2021; Gaudioso et al. 2021; Foysal and Gupta 2022; Rangel et al. 2022a; Hasan et al. 2023). Accordingly, the gut microbial communities of fish fed HIM differed from those of the control groups at the end of the present feeding experiment. Although no increase in species richness or biodiversity was observed, multivariate analysis based on unweighted and weighted UniFrac dissimilarity data revealed differences in response to feeding. Regardless of the diet, the three most frequently observed phyla were Firmicutes, Proteobacteria, and Fusobacteria. The presence of Fusobacteria instead of Actinobacteria, which are often reported in gilthead seabream (Huyben et al. 2020; Parma et al. 2020; Piazzon et al. 2020; Rimoldi et al. 2020; Panteli et al. 2021; Solé-Jiménez et al. 2021; Busti et al. 2024), is remarkable. In contrast to the previous studies conducted in RAS or flow-through systems (FTS), the current feeding trial took place in sea cages under environmental conditions similar to those of commercial seabream farming. Fish reared in sea cages are exposed to environmental conditions such as water temperature and salinity that can affect their gut microbiota. For example, the transition from freshwater to seawater, which is a common event in the life cycle of Atlantic salmon, has been shown to have profound effects on the host gut microbiota (Wang et al. 2021). In contrast, the gut microbiota of fish in an experimental-scale facility (RAS or FTS) could be influenced by bacterial communities in the rearing water, in biofilters (in RAS) and in biofilms on the tank surface, as well as by suspended solids (Bruno et al. 2023). Therefore, in addition to diet, different rearing conditions could also influence the gut microbiota of fish differently.

At the family level, gilthead seabream fed with HIM showed lower abundance of Fusobacteriaceae and higher abundance of Bacillaceae and Paenibacillaceae compared to controls. As mentioned above, an increase in the proportion of Bacillaceae has often been associated with the feeding of insect meal. Their increase is generally considered beneficial as this bacterial family contains several species with proven probiotic properties, such as *Bacillus subtilis* (Olmos et al. 2020). In contrast to previous data in rainbow trout, an 11% content of HI meal was not sufficient to increase the abundance of lactic acid bacteria in the gut of gilthead seabream (Terova et al. 2019). Unfortunately, very few studies have addressed the effects of feeding insect meal on the gut microbiota of gilthead seabream, especially in the context of novel formulations with low marine components. Furthermore, to our knowledge, none of these studies has been conducted on a farm scale in marine cages. Nevertheless, our results are in agreement with those of Busti et al. (2024), who found a significant enrichment of Bacillaceae and Paenibacillaceae with a concomitant reduction of LAB in insect-fed gilthead seabream. In contrast, Piazzon and colleagues (2022) found an increase in *Lactobacillus* genus in gilthead seabream fed FM-free diets containing 10% meal from HI, but in this case, 15% microbial biomass produced by fermentation of *Corynebacterium glutamicum* and *Methylococcus capsulatus* was also added to the diet. However, because this study evaluated the entire formulation and not the effects of each ingredient, it is not possible to relate this response specifically to the insect meal. In another study, the number of Fusobacteria in the gut microbiota of gilthead seabream increased, while the number of Firmicutes and Actinobacteria decreased in response to the replacement of FM with HIM (Panteli et al. 2021). In the same study, an increased proportion of Staphylococcaceae was found in gilthead seabream fed with HI meal.

At the genus level, *Oceanobacillus* and *Paenibacillus* have been detected exclusively in the guts of fish fed HIM. Recently, according to our data, a strong increase in the relative abundance of the genera *Oceanobacillus* and *Paenibacillus* was reported in gilthead seabream in response to the inclusion of 5% to 15% HI meal in the diet (Busti et al. 2024). *Oceanobacillus* is considered a probiotic bacterium, and its association with the ingestion of insect meal or exuviae has already been established in rainbow trout (Rimoldi et al. 2021, 2023; Biasato et al. 2022)..

*Oceanobacillus* is also known for its ability to produce various enzymes, antibiotics, and exopolysaccharides and is significantly positively correlated with sucrose production in rainbow trout (Zhao et al. 2023).

Although the application of *Paenibacillus* in aquaculture is limited, this genus shares several characteristics with Bacillus that make *Paenibacillus* a good probiotic candidate. It can produce antimicrobial compounds and volatile organic compounds, improve growth performance and immune status of fish, and degrade non-starch polysaccharides (Gupta et al. 2016; Chen et al. 2019; Amoah et al. 2020).

However, the most interesting ability of the genus *Paenibacillus* is its chitinolytic activity (Grady et al. 2016; Rangel et al. 2022a, b). The presence of chitinolytic bacteria is particularly important when feeding insect-derived ingredients to fish because they promote chitin digestion. In agreement with our results, the modulation of the microbiota in trout digesta by HI meal was closely associated with the presence of *Paenibacillus* (Rimoldi et al. 2021). In European seabass, however, consumption of diets enriched with HI exuviae, but not HI meal, resulted in an increase in this genus (from not detected to 5.5% in relative abundance) (Rangel et al. 2022a).

In contrast, the gut microbiota of gilthead seabream fed FM was dominated by the genus *Cetobacterium*, which belongs to the phylum Fusobacteria. This phylum is widely distributed in the gut of omnivorous and carnivorous freshwater fish, and within Fusobacterium, *Cetobacterium* is the major commensal genus that is dominant in several fish species (Ofek et al. 2021). A *Cetobacterium* species closely related to *C. somerae* has been isolated and characterized from the digestive tract of five freshwater fish species, demonstrating its ability to produce vitamin B12 (Tsuchiya et al. 2008). *C. somerae* is also involved in glucose homeostasis and carbohydrate metabolism in fish (Wang et al. 2021). In addition, the numerous genes in the genome of *C. somerae* related to protein digestion may indirectly explain the greater abundance of this genus in fish fed an animal diet compared to a plant diet (Hao et al. 2017). In contrast, proteins from plant ingredients, mainly soybean meal, are associated with a high intake of dietary fiber and indigestible polysaccharides. Consequently, carbohydrate-active bacteria such as the genus *Bacillus*, whose genomes contain members of the cellulase, hemicellulose, and pectinase families, are generally positively associated with plant-based diets (Serra et al. 2019).

However, predictive functional profiling of the microbial communities revealed no difference in carbohydrate or protein metabolism between the two feeding groups. PICRUSt analysis revealed that genes associated with motility, chemotaxis, and the two-component signal transduction system were significantly more abundant. In general, bacteria use chemotaxis to enhance the acquisition of high-quality nutrients in their environment, but it could also improve the efficiency of environmental colonization by motile bacteria, swarming, and biofilm formation (Colin et al. 2021). The two-component signal transduction system is instead involved in mediating the response of bacteria to a wide range of signals and stimuli, including nutrients (Laub and Goulian 2007). This result may be due to the higher proportion of indigestible carbohydrates such as chitin in the diet of HIM, which act as probiotics to stimulate the growth and/or activity of certain microorganisms, such as chemotactic bacteria from the Bacillaceae family, which are abundant in the guts of HIM.

Accordingly, the levels of volatile SCFAs, especially acetic and propionic acids, were higher in fecal samples from fish fed HIM. SCFAs, which include butyrate, are the main metabolites produced in the intestine by bacterial fermentation of dietary fiber, and they act as anti-inflammatory agents that play a key role in regulating the immune system of fish (Canani et al. 2011; Terova et al. 2016). They are the main product of bacterial fermentation of chitin produced by chitinolytic bacteria, such as *Paenibacillus*, which were present in greater abundance in the insect meal feeding group. Similarly, rainbow trout fed diets containing exuviae or shrimp meal had increased levels of acetic acid and propionic acid in feces compared to FM (Rimoldi et al. 2023). Inclusion of insect exuviae in the diet also increased butyrate content, a desirable effect that we were unfortunately unable to achieve in this experiment.

In conclusion, the present study represents one of the few on-farm experiments with gilthead seabream, one of the most important species for aquaculture in the Mediterranean. The results confirmed that HI is a suitable alternative to FM, even at 11%, which is in line with the aqua feed industry’s demand to reduce feed production costs. Overall, the data suggest that HI meal may provide benefits to gut health and microbiota profile by enhancing probiotic and chitinolytic bacterial genera, leading to improved utilization of nutrients with the production of SCFAs that are absorbed by the host, contributing to its energy metabolism.

### Supplementary Information

Below is the link to the electronic supplementary material.

Supplementary file1 (XLSX 11 KB)

Supplementary file2 (XLSX 14 KB)

## Data Availability

All raw sequencing data were submitted to the European Nucleotide Archive (EBI ENA) public database under accession code PRJEB64777.

## References

[CR1] Albrektsen S, Kortet R, Vilhelm Skov P, et al (2022) Future feed resources in sustainable salmonid production: a review. 10.1111/raq.12673

[CR2] Alfiko Y, Xie D, Astuti RT (2022). Insects as a feed ingredient for fish culture: status and trends. Aquac Fish.

[CR3] Amoah K, Qin-Cheng H, Xiao-Hui D et al (2020) *Paenibacillus polymyxa* improves the growth, immune and antioxidant activity, intestinal health, and disease resistance in *Litopenaeus vannamei* challenged with *Vibrio parahaemolyticus*. Aquaculture 518:734563. 10.1016/j.aquaculture.2019.734563

[CR4] Anedda R, Melis R, Palomba A (2023). Balanced replacement of fish meal with *Hermetia illucens* meal allows efficient hepatic nutrient metabolism and increases fillet lipid quality in gilthead seabream (*Sparus aurata*). Aquaculture.

[CR5] AOAC (2019). Official methods of analysis.

[CR6] Barragan-Fonseca KB, Dicke M, van Loon JJA (2017). Nutritional value of the black soldier fly (*Hermetia illucens L*.) and its suitability as animal feed - a review. J Insects as Food Feed.

[CR7] Barroso FG, de Haro C, Sánchez-Muros MJ (2014). The potential of various insect species for use as food for fish. Aquaculture.

[CR8] Belghit I, Liland NS, Waagbø R (2018). Potential of insect-based diets for Atlantic salmon (*Salmo salar*). Aquaculture.

[CR9] Belghit I, Liland NS, Gjesdal P (2019). Black soldier fly larvae meal can replace fish meal in diets of sea-water phase Atlantic salmon (*Salmo salar*). Aquaculture.

[CR10] Biasato I, Chemello G, Oddon SB (2022). *Hermetia illucens* meal inclusion in low-fishmeal diets for rainbow trout (*Oncorhynchus mykiss*): effects on the growth performance, nutrient digestibility coefficients, selected gut health traits, and health status indices. Anim Feed Sci Technol.

[CR11] Bolyen E, Rideout JR, Dillon MR (2019). Reproducible, interactive, scalable and extensible microbiome data science using QIIME 2. Nat Biotechnol.

[CR12] Bonelli M, Bruno D, Brilli M (2020). Black soldier fly larvae adapt to different food substrates through morphological and functional responses of the midgut. Int J Mol Sci.

[CR13] Borrelli L, Varriale L, Dipineto L (2021). Insect derived lauric acid as promising alternative strategy to antibiotics in the antimicrobial resistance scenario. Front Microbiol.

[CR14] Bosi A, Banfi D, Moroni F (2021). Effect of partial substitution of fishmeal with insect meal (*Hermetia illucens*) on gut neuromuscular function in Gilthead sea bream (*Sparus aurata*). Sci Rep.

[CR15] Bruni L, Belghit I, Lock E (2020). Total replacement of dietary fish meal with black soldier fly (*Hermetia illucens* ) larvae does not impair physical, chemical or volatile composition of farmed Atlantic salmon ( *Salmo salar* L.). J Sci Food Agric.

[CR16] Bruno A, Sandionigi A, Panio A (2023). Aquaculture ecosystem microbiome at the water-fish interface: the case-study of rainbow trout fed with *Tenebrio molitor* novel diets. BMC Microbiol.

[CR17] Busti S, Bonaldo A, Candela M (2024). Hermetia illucens larvae meal as an alternative protein source in practical diets for gilthead sea bream (*Sparus aurata*): a study on growth, plasma biochemistry and gut microbiota. Aquaculture.

[CR18] Caballero MJ, Izquierdo MS, Kjørsvik E (2004). Histological alterations in the liver of sea bream, *Sparus aurata L*., caused by short- or long-term feeding with vegetable oils. Recovery of normal morphology after feeding fish oil as the sole lipid source. J Fish Dis.

[CR19] Camargo MMP, Martinez CBR (2007). Histopathology of gills, kidney and liver of a Neotropical fish caged in an urban stream. Neotrop Ichthyol.

[CR20] Canani RB, Di CM, Leone L (2011). Potential beneficial effects of butyrate in intestinal and extraintestinal diseases. World J Gastroenterol.

[CR21] Carvalho M, Torrecillas S, Montero D (2023). Insect and single-cell protein meals as replacers of fish meal in low fish meal and fish oil diets for gilthead sea bream (*Sparus aurata*) juveniles. Aquaculture.

[CR22] Ceccotti C, Bruno D, Tettamanti G (2022). New value from food and industrial wastes – bioaccumulation of omega-3 fatty acids from an oleaginous microbial biomass paired with a brewery by-product using black soldier fly (*Hermetia illucens*) larvae. Waste Manag.

[CR23] Chemello G, Renna M, Caimi C (2020). Partially defatted *Tenebrio molitor* larva meal in diets for grow-out rainbow trout, *Oncorhynchus mykiss* (Walbaum): effects on growth performance, diet digestibility and metabolic responses. Animals.

[CR24] Chen SW, Liu CH, Hu SY (2019). Dietary administration of probiotic *Paenibacillus ehimensis* NPUST1 with bacteriocin-like activity improves growth performance and immunity against *Aeromonas hydrophila* and *Streptococcus iniae* in Nile tilapia (*Oreochromis niloticus*). Fish Shellfish Immunol.

[CR25] Chlebicz-Wójcik A, Śliżewska K (2020). The effect of recently developed synbiotic preparations on dominant fecal microbiota and organic acids concentrations in feces of piglets from nursing to fattening. Animals.

[CR26] Colin R, Ni B, Laganenka L, Sourjik V (2021). Multiple functions of flagellar motility and chemotaxis in bacterial physiology. FEMS Microbiol Rev.

[CR27] Cullere M, Woods MJ, van Emmenes L (2019). *Hermetia illucens* larvae reared on different substrates in broiler quail diets: effect on physicochemical and sensory quality of the quail meat. Animals.

[CR28] Dalle Zotte A, Singh Y, Michiels J, Cullere M (2019). Black soldier fly (*Hermetia Illucens*) as dietary source for laying quails: live performance, and egg physico-chemical quality, sensory profile and storage stability. Animals.

[CR29] Di Rosa AR, Caccamo L, Pansera L (2023). Influence of *Hermetia illucens* larvae meal dietary inclusion on growth performance, gut histological traits and stress parameters in *Sparus aurata*. Animals.

[CR30] Dong Y, Wei Y, Wang L (2023). Dietary n-3/n-6 polyunsaturated fatty acid ratio modulates growth performance in spotted seabass (*Lateolabrax maculatus*) through regulating lipid metabolism, hepatic antioxidant capacity and intestinal health. Anim Nutr.

[CR31] Escaffre AM, Kaushik S, Mambrini M (2007). Morphometric evaluation of changes in the digestive tract of rainbow trout (*Oncorhynchus mykiss*) due to fish meal replacement with soy protein concentrate. Aquaculture.

[CR32] Ewald N, Vidakovic A, Langeland M (2020). Fatty acid composition of black soldier fly larvae (*Hermetia illucens*) – possibilities and limitations for modification through diet. Waste Manag.

[CR33] Fabrikov D, Vargas-García MdC, Barroso FG (2021). Effect on intermediary metabolism and digestive parameters of the high substitution of fishmeal with insect meal in *Sparus aurata* feed. Insects.

[CR34] FAO (2020) The State of World Fisheries and Aquaculture 2020. In brief. State World Fish Aquac 2020 Br. 10.4060/CA9231EN

[CR35] Ferramosca A, Zara V (2014). Modulation of hepatic steatosis by dietary fatty acids. World J Gastroenterol.

[CR36] Foysal MJ, Gupta SK (2022). A systematic meta-analysis reveals enrichment of Actinobacteria and Firmicutes in the fish gut in response to black soldier fly (*Hermetica illucens*) meal-based diets. Aquaculture.

[CR37] Gasco L, Biasato I, Dabbou S (2019). Aanimals fed insect-based diets: state-of-the-art on digestibility, performance and product quality. Animals.

[CR38] Gaudioso G, Marzorati G, Faccenda F (2021). Processed animal proteins from insect and poultry by-products in a fish meal-free diet for rainbow trout: impact on intestinal microbiota and inflammatory markers. Int J Mol Sci.

[CR39] Grady EN, MacDonald J, Liu L (2016). Current knowledge and perspectives of *Paenibacillus*: a review. Microb Cell Fact.

[CR40] Gupta A, Gupta P, Dhawan A (2016). *Paenibacillus polymyxa* as a water additive improved immune response of *Cyprinus carpio* and disease resistance against *Aeromonas hydrophila*. Aquac Reports.

[CR41] Hammer DAT, Ryan PD, Hammer Ø, Harper DAT (2001) Past: Paleontological Statistics Software Package for education and data analysis. Palaeontol Electron 4:178. https://palaeo-electronica.org/2001_1/past/past.pdf. Accessed 30 Aug 2023

[CR42] Hao YT, Wu SG, Xiong F (2017). Succession and fermentation products of grass carp (*Ctenopharyngodon idellus*) hindgut microbiota in response to an extreme dietary shift. Front Microbiol.

[CR43] Hasan I, Rimoldi S, Saroglia G, Terova G (2023). Sustainable fish feeds with insects and probiotics positively affect freshwater and marine fish gut microbiota. Animals.

[CR44] Henry M, Gasco L, Piccolo G, Fountoulaki E (2015). Review on the use of insects in the diet of farmed fish: past and future. Anim Feed Sci Technol.

[CR45] Hoc B, Genva M, Fauconnier ML, et al (2020) About lipid metabolism in *Hermetia illucens* (L. 1758): on the origin of fatty acids in prepupae. Sci Rep 10. 10.1038/S41598-020-68784-8 10.1038/s41598-020-68784-8 PMC7368053 32680992

[CR46] Hosseindoust A, Ha SH, Mun JY, Kim JS (2023). Quality characteristics of black soldier flies produced by different substrates. Insects.

[CR47] Huyben D, Rimoldi S, Ceccotti C, Montero D, Betancor M, Iannini F, Terova G (2020) Effect of dietary oil from *Camelina sativa* on the growth performance, fillet fatty acid profile and gut microbiome of gilthead Sea bream (*Sparus aurata*). PeerJ 8:e10430. 10.7717/peerj.10430 10.7717/peerj.10430 PMC7733328 33354421

[CR48] Huyben D, Vidaković A, Werner Hallgren S, Langeland M (2019). High-throughput sequencing of gut microbiota in rainbow trout (*Oncorhynchus mykiss*) fed larval and pre-pupae stages of black soldier fly (*Hermetia illucens*). Aquaculture.

[CR49] Karapanagiotidis IT, Neofytou MC, Asimaki A (2023). Fishmeal replacement by full-fat and defatted
* Hermetia illucens
* prepupae meal in the diet of gilthead seabream (*Sparus aurata*). Sustainability.

[CR50] Kumar P, Lee JH, Beyenal H, Lee J (2020). Fatty acids as antibiofilm and antivirulence agents. Trends Microbiol.

[CR51] Langille MGI, Zaneveld J, Caporaso JG (2013). Predictive functional profiling of microbial communities using 16S rRNA marker gene sequences. Nat Biotechnol.

[CR52] Laub MT, Goulian M (2007). Specificity in two-component signal transduction pathways. Annu Rev Genet.

[CR53] Li Y, Kortner TM, Chikwati EM (2020). Total replacement of fish meal with black soldier fly (*Hermetia illucens*) larvae meal does not compromise the gut health of Atlantic salmon (*Salmo salar*). Aquaculture.

[CR54] Li Y, Bruni L, Jaramillo-Torres A (2021). Differential response of digesta- and mucosa-associated intestinal microbiota to dietary insect meal during the seawater phase of Atlantic salmon. Anim Microbiome.

[CR55] Lock ER, Arsiwalla T, Waagbø R (2016). Insect larvae meal as an alternative source of nutrients in the diet of Atlantic salmon (*Salmo salar*) postsmolt. Aquac Nutr.

[CR56] Lozupone C, Knight R (2005). UniFrac: a new phylogenetic method for comparing microbial communities. Appl Environ Microbiol.

[CR57] Lozupone CA, Hamady M, Kelley ST, Knight R (2007). Quantitative and qualitative beta diversity measures lead to different insights into factors that structure microbial communities. Appl Environ Microbiol.

[CR58] Mastoraki M, Katsika L, Enes P (2022). Insect meals in feeds for juvenile gilthead seabream (*Sparus aurata*): effects on growth, blood chemistry, hepatic metabolic enzymes, body composition and nutrient utilization. Aquaculture.

[CR59] Meneguz M, Schiavone A, Gai F (2018). Effect of rearing substrate on growth performance, waste reduction efficiency and chemical composition of black soldier fly (*Hermetia illucens*) larvae. J Sci Food Agric.

[CR60] Nawaz A, Bakhsh javaid A, Irshad S,  (2018). The functionality of prebiotics as immunostimulant: evidences from trials on terrestrial and aquatic animals. Fish Shellfish Immunol.

[CR61] Naya-Català F, do Vale Pereira  G, Piazzon MC (2021). Cross-talk between intestinal microbiota and host gene expression in gilthead sea bream (*Sparus aurata*) juveniles: insights in fish feeds for increased circularity and resource utilization. Front Physiol.

[CR62] Ofek T, Lalzar M, Laviad-Shitrit S (2021). Comparative study of intestinal microbiota composition of six edible fish species. Front Microbiol.

[CR63] Olmos J, Acosta M, Mendoza G, Pitones V (2020). *Bacillus subtilis*, an ideal probiotic bacterium to shrimp and fish aquaculture that increase feed digestibility, prevent microbial diseases, and avoid water pollution. Arch Microbiol.

[CR64] Oteri M, Di Rosa AR, Lo Presti V (2021). Black soldier fly larvae meal as alternative to fish meal for aquaculture feed. Sustain.

[CR65] Oteri M, Chiofalo B, Maricchiolo G, Toscano G, Nalbone L, Lo Presti V, Di Rosa AR (2022). Black Soldier fly larvae meal in the diet of gilthead sea bream: effect on chemical and microbiological quality of filets. Front Nutr.

[CR66] Palomba A, Melis R, Biosa G (2022). On the compatibility of fish meal replacements in aquafeeds for rainbow trout. A combined metabolomic, proteomic and histological study. Front Physiol.

[CR67] Panteli N, Mastoraki M, Lazarina M (2021). Configuration of gut microbiota structure and potential functionality in two teleosts under the influence of dietary insect meals. Microorganisms.

[CR68] Parks DH, Tyson GW, Hugenholtz P, Beiko RG (2014). Genome analysis STAMP: statistical analysis of taxonomic and functional profiles. Bioinformatics.

[CR69] Parma L, Pelusio NF, Gisbert E (2020). Effects of rearing density on growth, digestive conditions, welfare indicators and gut bacterial community of gilthead sea bream (Sparus aurata, L. 1758) fed different fishmeal and fish oil dietary levels. Aquaculture.

[CR70] Piazzon MC, Naya-Català F, Perer E (2020). Genetic selection for growth drives differences in intestinal microbiota composition and parasite disease resistance in gilthead sea bream. Microbiome.

[CR71] Piazzon MC, Naya-Català F, Pereira GV (2022). A novel fish meal-free diet formulation supports proper growth and does not impair intestinal parasite susceptibility in gilthead sea bream (*Sparus aurata*) with a reshape of gut microbiota and tissue-specific gene expression patterns. Aquaculture.

[CR72] Quast C, Pruesse E, Yilmaz P (2013). The SILVA ribosomal RNA gene database project: improved data processing and web-based tools. Nucleic Acids Res.

[CR73] Randazzo B, Zarantoniello M, Cardinaletti G (2021). *Hermetia illucens* and poultry by-product meals as alternatives to plant protein sources in gilthead seabream (*Sparus aurata*) diet: a multidisciplinary study on fish gut status. Animals.

[CR74] Rangel F, Enes P, Gasco L (2022). Differential modulation of the European sea bass gut microbiota by distinct insect meals. Front Microbiol.

[CR75] Rangel F, Santos RA, Monteiro M (2022). Isolation of chitinolytic bacteria from european sea bass gut microbiota fed diets with distinct insect meals. Biology (basel).

[CR76] Rašković BS, Stanković MB, Marković ZZ, Poleksić VD (2011). Histological methods in the assessment of different feed effects on liver and intestine of fish. J Agric Sci.

[CR77] Rimoldi S, Gliozheni E, Ascione C (2018). Effect of a specific composition of short- and medium-chain fatty acid 1-Monoglycerides on growth performances and gut microbiota of gilthead sea bream (*Sparus aurata*). PeerJ.

[CR78] Rimoldi S, Gini E, Iannini F (2019). The effects of dietary insect meal from *Hermetia illucens
* prepupae on autochthonous gut microbiota of rainbow trout (* Oncorhynchus mykiss*). Animals.

[CR79] Rimoldi S, Gini E, Koch JFA (2020). Effects of hydrolyzed fish protein and autolyzed yeast as substitutes of fishmeal in the gilthead sea bream (*Sparus aurata*) diet, on fish intestinal microbiome. BMC Vet Res.

[CR80] Rimoldi S, Antonini M, Gasco L (2021). Intestinal microbial communities of rainbow trout (*Oncorhynchus mykiss*) may be improved by feeding *a Hermetia illucens* meal/low-fishmeal diet. Fish Physiol Biochem.

[CR81] Rimoldi S, Ceccotti C, Brambilla F (2023). Potential of shrimp waste meal and insect exuviae as sustainable sources of chitin for fish feeds. Aquaculture.

[CR82] Ripps H, Shen W (2012). Review: Taurine: a “very essential” amino acid. Mol Vis.

[CR83] Rodrigues S, Antunes SC, Nunes B, Correia AT (2017). Histological alterations in gills and liver of rainbow trout (*Oncorhynchus mykiss*) after exposure to the antibiotic oxytetracycline. Environ Toxicol Pharmacol.

[CR84] Sahoo A, Swain SS, Behera A, Sahoo G (2021). Antimicrobial peptides derived from insects offer a novel therapeutic option to combat biofilm: a review. Front Microbiol.

[CR85] Serra CR, Almeida EM, Guerreiro I (2019). Selection of carbohydrate-active probiotics from the gut of carnivorous fish fed plant-based diets. Sci Rep.

[CR86] Skrivanova E, Marounek M, Benda V, Brezina P (2006). Susceptibility of *Escherichia coli, Salmonella sp*. and Clostridium perfringens to organic acids and monolaurin. Vet-Med Czech.

[CR87] Sogari G, Amato M, Biasato I (2019). The potential role of insects as feed: a multi-perspective review. Animals.

[CR88] Solé-Jiménez P, Naya-Català F, Piazzon MC (2021). Reshaping of gut microbiotain gilthead sea bream fed microbial and processed animal proteins as the main dietary protein source. Front Mar Sci.

[CR89] Somayeh SM, Jiun Z, Loh Y (2020). A review on insect meals in aquaculture: the immunomodulatory and physiological effects. Int Aquat Res.

[CR90] Spranghers T, Michiels J, Vrancx J (2018). Gut antimicrobial effects and nutritional value of black soldier fly (*Hermetia illucens L*.) prepupae for weaned piglets. Anim Feed Sci Technol.

[CR91] Terova G, Díaz N, Rimoldi S (2016). Effects of sodium butyrate treatment on histone modifications and the expression of genes related to epigenetic regulatory mechanisms and immune response in European Sea Bass (*Dicentrarchus Labrax*) fed a plant-based diet. PLoS ONE.

[CR92] Terova G, Rimoldi S, Ascione C (2019). Rainbow trout (*Oncorhynchus mykiss*) gut microbiota is modulated by insect meal from *Hermetia illucens* prepupae in the diet. Rev Fish Biol Fish.

[CR93] Terova G, Gini E, Gasco L (2021). Effects of full replacement of dietary fishmeal with insect meal from *Tenebrio molitor* on rainbow trout gut and skin microbiota. J Anim Sci Biotechnol.

[CR94] Tsuchiya C, Sakata T, Sugita H (2008). Novel ecological niche of *Cetobacterium somerae*, an anaerobic bacterium in the intestinal tracts of freshwater fish. Lett Appl Microbiol.

[CR95] van Huis A (2020). Insects as food and feed, a new emerging agricultural sector: a review. J Insects as Food Feed.

[CR96] Wang A, Zhang Z, Ding Q (2021). Intestinal *Cetobacterium* and acetate modify glucose homeostasis via parasympathetic activation in zebrafish. Gut Microbes.

[CR97] Weththasinghe P, Rocha SDC, Øyås O (2022). Modulation of Atlantic salmon (*Salmo salar*) gut microbiota composition and predicted metabolic capacity by feeding diets with processed black soldier fly (*Hermetia illucens*) larvae meals and fractions. Anim Microbiome.

[CR98] Zarantoniello M, de Oliveira AA, Sahin T (2023). Enhancing rearing of European seabass (*Dicentrarchus labrax*) in aquaponic systems: investigating the effects of enriched black soldier fly (*Hermetia illucens*) prepupae meal on fish welfare and quality traits. Animals.

[CR99] Zhao C, Men X, Dang Y (2023). Probiotics mediate intestinal microbiome and microbiota-derived metabolites regulating the growth and immunity of rainbow trout (*Oncorhynchus mykiss*). Microbiol Spectr.

